# Comparative toxicity of ionic and nanoparticulate zinc in the species *Cymodoce truncata*, *Gammarus aequicauda* and *Paracentrotus lividus*

**DOI:** 10.1007/s11356-021-13712-0

**Published:** 2021-04-07

**Authors:** Ermelinda Prato, Adele Fabbrocini, Giovanni Libralato, Luciana Migliore, Isabella Parlapiano, Raffaele D’Adamo, Alice Rotini, Loredana Manfra, Giusy Lofrano, Federica Carraturo, Marco Trifuoggi, Francesca Biandolino

**Affiliations:** 1 CNR-IRSA National Research Council – Water Research Institute, Taranto, Italy; 2 CNR-ISMAR (National Research Council – Institute of Marine Sciences, Naples, Italy; 3 Department of Biology, University of Naples Federico II, Complesso Universitario di Monte Sant’Angelo, Via Cinthia 21, 80126 Naples, Italy; 4 Department of Biology, Tor Vergata University of Rome, Rome, Italy; 5 Institute for Environmental Protection and Research (ISPRA), Rome, Italy; 6 Department of Marine Biotechnology, Stazione Zoologica Anton Dohrn, Naples, Italy; 7 Centro Servizi Metrologici e Tecnologici Avanzati (CeSMA), Complesso Universitario di Monte Sant’Angelo, Via Cinthia 21, 80126 Naples, Italy; 8 Department of Chemical Sciences, University of Naples Federico II, Complesso Universitario di Monte Sant’Angelo, Via Cinthia 21, 80126 Naples, Italy

**Keywords:** Nano-ecotoxicology, Zinc oxide nanoparticles, Zinc sulphate, Crustaceans, Sea urchins

## Abstract

Due to the continuous development, production and consumption of nanoparticles (NPs), their release, fate and effects in marine coastal environment can represent a major concern. The aim of this study was to evaluate the toxicity of ZnO nanoparticles (ZnO NPs) and compare it to bulk ZnSO_4_ on three macroinvertebrates: the isopod *Cymodoce truncata* (i.e. used for the first time in ecotoxicology), the amphipod *Gammarus aequicauda* and the sea urchin *Paracentrotus lividus*. This study showed concentration- and time-dependent relationships for all biological models for both ZnO NPs and ZnSO_4_. Both Zn forms elicited high toxicity to *G. aequicauda* and *C. truncata juveniles*, but ZnO NPs induced comparable responses to both species (96h-LC_50_ = 0.30 and 0.37 mg/L for *G. aequicauda* and *C. truncata*, respectively; *p* > 0.05), while differences were found after ZnSO_4_ exposure (96h-LC_50_ = 0.28 and 0.63 mg/L, respectively; *p* < 0.05). ZnO NPs generated sub-lethal effects on *P. lividu*s embryos (72h-EC_50_ = 0.04 (0.03, 0.05) mg/L), not significantly different from ZnSO_4_ ones (72h-EC_50_ = 0.06 (0.05, 0.07) mg/L). Effects of ZnO NPs were similar to existing literature data for other testing species. *C. truncata* can be considered as a promising new biological model in (nano)ecotoxicology.

**Supplementary Information:**

The online version contains supplementary material available at 10.1007/s11356-021-13712-0.

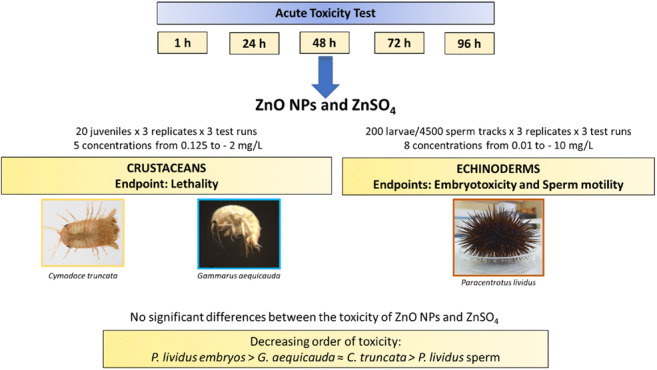

## Introduction

The production and use of engineered metal oxide nanoparticles (NPs) exponentially increased in the last decade because of their unique properties (small size, large surface area, surface reactivity, charge, shape, media interactions) that make them suitable for a wide variety of applications. These include the so-called environmentally friendly nanotechnologies, like commercial products for domestic and healthcare use, and constitute products for waste remediation, fuel and energy production (Lofrano et al. 2017). Among them, zinc oxide nanoparticles (ZnO NPs) are the third highest used metal-containing nanomaterials with an estimated annual global production ranging from 550 to 1600 tons/year (Peng et al. 2017; Piccinno et al. 2012; Sun et al. 2014). Their introduction in different materials demonstrated to provide a better alternative in many commercial processes by producing more durable ceramics, transparent solar filter blocking infrared and ultraviolet radiation and reducing the amount of ZnO in the production of paints, plastic, pigments and catalysts (Nemček et al. 2020; Piccinno et al. 2012). ZnO NPs are also useful in biomedical research since they exhibited selective toxicity for human cancer cell lines and thus are reported as potential ‘tools’ in chemotherapy (Jiang et al. 2018). ZnO NPs may also be toxic for some microorganisms, making them potential antibacterial, antifungal and antiviral agents, so they are utilized as disinfectant in aquaculture (Khosravi-Katuli et al. 2017); however, an opposite effect may occasionally happen when ZnO NPs enter wastewater streams at treatment plants and inhibit bacterial activities in the activated sludge, affecting negatively treatment efficiency (Tan et al. 2015).

Zinc oxide NPs have been deemed as ‘extremely toxic’ to aquatic organisms (Kahru and Dubourguier 2010), their toxicity being frequently imputed to the dissolution of ZnO NPs into toxic and bioavailable zinc forms (i.e. Zn ions free or weakly complexed with inorganic or organic ligands) (Aruoja et al. 2009; Yung et al. 2015). Moreover, ZnO NPs toxicity may be also due to free radical formation and induction of oxidative stress with the generation of reactive oxygen species (ROS), able to induce DNA (Bai et al. 2010; Zhao et al. 2013), protein, lipid and tissue (Khosravi-Katuli et al. 2018) damage. In addition, the direct interaction between NPs and cells or organisms may cause mechanical damage like shortening and collapsing of gill secondary lamellae in adult fish (Khosravi-Katuli et al. 2018). NPs can also bind to different ligands and acquire several different coatings often within a small period of time, which makes measuring them even harder especially in terms of shape, size and concentration (Diegoli et al. 2008; Nowack and Bucheli 2007; Tiede et al. 2008). Nevertheless, according to the available data, the predicted average environmental concentration of ZnO NPs in European surface waters is of 0.1 mg/L (Sun et al. 2014; Tan et al. 2015).

The presence of NPs in marine systems, in particular ZnO NPs, is a great concern, and the optimization of easy-to-perform and quick-response tools specifically tailored to evaluate their effects represents a mandatory issue. The ecotoxicological effects of ZnO NPs have been investigated by acute toxicity tests on a wide number of freshwater organisms, including bacteria (Sirelkhatim et al. 2015), algae (Franklin et al. 2007), crustaceans (Prato et al. 2020; Vimercati et al. 2020; Wang et al. 2009) and fish (Khosravi-Katuli et al. 2018). Studies on ZnO NPs ecotoxicity have been performed also on marine organisms belonging to different taxonomic groups, including microalgae, molluscs, crustaceans and fishes (Abdel-Khalek 2015; Fairbairn et al. 2011; Minetto et al. 2016; Prato et al. 2020; Schiavo et al. 2018; Vimercati et al. 2020; Wong et al. 2010).

Crustaceans are among the most common model organisms in acute ecotoxicity assays. They play a key role in the marine food webs and their energy flow, acting as both prey and predator at various trophic levels, frequently in the sedimentary facies, thus being a potential target of NPs (Prato et al. 2020). Among them, members of the Gammaridae family are often utilized to assess ecotoxicity due to their sensitivity and availability in the area, such as *Gammarus aequicauda* (Prato et al. 2013, 2015), which is why we chose it for the present study. This species plays an important role both in the trophic web and in the organic matter recycling of underlying sediment (Cottiglia et al. 1983). Isopods also are important keystone species in marine ecosystems, but their sensitivity to contaminants is hitherto little studied (Prato et al. 2006), although they are relevant models in soil ecotoxicology. No ecotoxicological data exists on *Cymodoce truncata* (Leach, 1814), so this is the first study using juveniles of *C. truncata* for toxicity testing. It usually thrives in coastal marine or brackish waters inhabiting seaweed fronds (i.e. haloresistant mesograzer) between 5 and 80 cm depth.

Sea urchins are a commonly used model organisms for laboratory research; their gametes and embryos have been exposed to a wide range of environmental contaminants, and the consequent alterations at the morphological, physiological and gene expression levels have been studied (Fabbrocini and D’Adamo 2011; Ruocco et al. 2017). Thus, sea urchins are well-established models in ecotoxicological studies (ASTM 2004), and among the Mediterranean species, *Paracentrotus lividus* has been standardized for ecotoxicity testing since a long time (Arizzi Novelli et al. 2006) involving alterations of sperm motility and early embryo–larval development as endpoints.

The aim of this research was to compare the toxic effects of ZnO NPs with those of the soluble zinc salt, ZnSO_4_, in acute ecotoxicological tests with two crustaceans (*C. truncata* and *G. aequicauda*) and the echinoderm *P. lividus*. These species can be considered of high interest in nanoecotoxicology: both *C. truncata* and *G. aequicauda* are bottom filter feeders and deposit feeders; thus, they can be potentially at high risk of exposure in nature, due to the tendency of NPs to aggregate in seawater settling down and accumulating in sediment (Garner and Keller 2014); equally, *P. lividus*, releasing gametes in the water column, where fertilization and larval development occur (Boudouresque and Verlaque 2013), may be exposed to NP contamination in these pivotal phases of the biological cycle.

## Materials and methods

### Preparation and characterization of testing media

Water-dispersed ZnO NPs (20 wt%, purity of 99.95%) (S1) were purchased from US Research Nanomaterials, Inc. (Houston, USA) with a nominal particle size of 30–40 nm. A stock suspension (1000 mg/L) of ZnO NPs (S2) was prepared in 0.22-μm filtered ultrapure water from S1 and sonicated for 15 min in a ultrasonic water bath (305 W, 50–60 Hz; Soltec Ultrasonic Baths) and stored in the dark at 4 °C for 15 min. Final testing suspensions were prepared in filtered (GF/C Whatman, 0.22 μm) natural seawater (FNSW) collected from the Mar Grande of Taranto (Ionian Sea, Italy; 40° 25′ 0.1″ N, 17° 14′ 24″ E; pH 8.0 ± 0.1).

A stock solution of ZnSO_4_ (Sigma-Aldrich, Germany, ≥ 99.9%) (1000 mg/L) was prepared by dissolving the toxicant in 0.22-μm FNSW, stirring until complete dissolution and stored in the dark at 4 °C. Final testing solution concentrations were prepared in FNSW at the same nominal concentrations of ZnO NP suspensions.

The size distribution of Zn in both solutions and suspensions was carried out using a dynamic light scattering (DLS) composed by a Photocor compact goniometer, a SMD 6000 Laser Quantum 50-mW light source operating at 5325 Å and a photo-multiplier tube (PMT, PMT-120-OP/B) and correlator (Flex02-01D) purchased from Correlator.com. The hydrodynamic radius was evaluated through the Stokes-Einstein equation (Mangiapia et al. 2013). Each measurement was performed in triplicate. The samples (1 mg/L) were prepared from a starting solution (1 g/L) produced with 0.22-μm FNSW and sonicated at 100 W for three cycles of 5 min each, leaving to rest the sample for 5 min between two cycles. Finally, each sample was left to equilibrate in the instrument for 15 min before the measurement. Exclusively in the case of ZnO NPs, size distribution was followed from 15 min after sonication until 48 h of ageing in FNSW.

The size of the ZnO NPs was evaluated by mean of transmission electron microscopy (Lattemann and Höpner 2008) using a Philips EM 208S with an accelerating voltage of 100 kV. All details about ZnO NPs’ characterization have been already published in (Prato et al. 2020).

The concentration of Zn from ZnSO_4_ and ZnO NP solution/suspensions was quantified by inductively coupled plasma mass spectrometry (Aurora M90 Bruker, USA) (Table 1). The detection (LOD = 0.02 μg/L) and quantification (LOQ = 0.04 μg/L) limits were calculated using the method of blank variability for each investigated metal (EPA 2014).

**Table 1:** Nominal and measured total Zn concentration in ZnO NPs and ZnSO_4_ testing suspensions/solutions and control (filtered natural seawater, FN SW; as mean values ± standard deviation, *n* = 3 per testing suspensions/solutions and seawater)

Zn concentrations (mg/L)	*C. truncata* and *G. aequicauda*		*P. lividus*	
	Nominal	Measured	Nominal	Measured
FNSW	-	0.001 ± 0.00	-	0.001 ± 0.00
ZnO NPs	0.12	0.11 ± 0.00	0.01	0.02 ± 0.00
	0.25	0.23 ± 0.05	0.05	0.06 ± 0.00
	0.5	0.48 ± 0.09	0.1	0.11 ± 0.00
	1	0.85 ± 0.17	0.2	0.16 ± 0.03
	2	1.26 ± 0.25	0.5	0.48 ± 0.09
			1	0.85 ± 0.17
ZnSO_4_	0.12	0.15 ± 0.05	0.01	0.02 ± 0.00
	0.25	0.28 ± 0.05	0.05	0.04 ± 0.00
	0.5	0.52 ± 0.10	0.1	0.15 ± 0.05
	1	0.93 ± 0.08	0.2	0.30 ± 0.07
	2	1.73 ± 0.35	0.5	0.52 ± 0.10
			1	0.93 ± 0.08

### Chemical analysis and NP characterization

The nominal and measured concentrations of Zn in testing suspensions/solutions are reported in Table 1, for ZnO NPs’ and ZnSO_4_ tests. The NP characterization, as already reported by (Prato et al. 2020), demonstrated that the hydrodynamic radius distribution for ZnSO_4_ solutions was 950 ± 50 nm, while in ZnO NP suspensions, two peaks (at 150 ± 20 nm and 640 ± 30 nm) were found to be related to the production of aggregates. Nevertheless, TEM analysis showed NPs ranging between 10 and 20 nm. According to Prato et al. (2020), the average dissolution of ZnO NPs was 46% (ranging from 22 up to 68%) similar to (Wong et al. 2010) (3.7 mg Zn/L), confirming the suitability of the prepared suspensions/solutions.

### Toxicity testing

#### *C. truncata* and *G. aequicauda*

Isopods were collected from an unpolluted area located in the Taranto Gulf (Ionian Sea, Southern Italy; 40° 49′ 63″ N, 17° 32′ 35″ E), used as a local reference site because of the low levels of anthropogenic pollution (unpublished data). Juveniles were collected from the macroalgae *Chaetomorpha linum* and *Ulva sp*., transported to the laboratory and placed in culture tanks filled with FNSW (0.45 μm and 36 ± 2 PSU), which was continuously aerated and maintained at 18 ± 2 °C with a 12:12 h light/dark cycle. Both species were acclimated to the above experimental conditions for at least 7–10 days after field collection. During the acclimation period, *C. truncata* and *G. aequicauda* were fed weekly with the seaweed *Chaetomorpha linum*, and benthic microalgae *Phaeodactylum tricornutum* were added to the tanks to integrate the organic matter present in the native sediment.

Juveniles of *C. truncata* and *G. aequicauda* were utilized in a semi-static acute toxicity test (i.e. suspensions/solutions were renewed after 48 h). They were exposed for 96 h in triplicate to 0.12, 0.25, 0.50, 1.00 and 2.00 mg/L (nominal concentrations) of test suspensions/solutions, including a negative control (FNSW) (Table 1). For isopod species, twenty individuals per concentration were randomly selected from the culture tanks and added to a 250-mL glass beaker containing 200 mL of test suspension/solution. Test species were not fed during the testing period. Water quality parameters (temperature, pH, dissolved oxygen and salinity) were measured at the beginning and at the end of each test to check quality assurance and quality control (ISO 16712:^2005^). Mortality was checked daily, and dead animals were removed; missing animals were considered dead; apparently dead individuals were considered living if movement was exhibited after gentle stimulation. At the end of the experiments, the surviving organisms were counted. The mortality threshold value was 10% in negative controls for the above toxicity tests (USEPA 1994).

#### P. lividus

Adult sea urchins (35 to 45 mm in diameter without spines) were hand-collected with the aid of scuba diving equipment along the southern Adriatic coast of Italy (Termoli, CB; 41° 54′ N, 16° 10′ E). Collected specimens were placed in a cooler and carried to the laboratory under moist conditions for no more than 2 h. Sea urchins were then placed in a recirculating aquarium and reared as described in (Fabbrocini and D’Adamo 2011) until gamete collection. Sea urchins were induced to spawn by injection of 1 mL of KCl 0.5 M. Gametes were observed by microscope, and the best male and female samples were selected for the pools (at least three individuals for each pool). The sperm pools were dry stored at 4 °C, while the eggs were gently washed in FNSW and kept at 18 °C. Embryotoxicity test was performed within 1 h of gamete collection. The embryotoxicity test was carried out as described in (Fabbrocini and D’Adamo 2011). Fertilized eggs were incubated at a final concentration of 200 eggs/mL in 10-ml polystyrene multiwell plates containing the test solutions (0.01, 0.05, 0.10, 0.20, 0.50, 1.00 mg/L of ZnO NPs and ZnSO_4_ in FNSW), until plutei larvae were obtained (72 h, 18 °C, in the dark). Samples were preserved in concentrated buffered formalin, and the percentages of normal developed plutei (NPL) were scored by observing 200 larvae for each sample. FNSW was used as a negative control, while scaled concentrations of copper were used for the positive control. The effects of ZnO NPs were evaluated on sperm cell motility according to the MOT-test (Fabbrocini et al. 2010). Sperm samples were diluted at a ratio of 1:1000 in 0.01, 0.05, 0.10, 0.20, 0.50, 1.00, 5.0 and 10.0 mg/L of ZnO NPs and ZnSO_4_ in FNSW and incubated for 60 min at 18 °C. On dilution and after incubation, sperm motility was evaluated by computerized motion analysis system, the Sperm Class Analyzer® (SCA, Microptic, s.l., Spain). The following motion parameters were assessed: (a) total motile sperm (TM), as the percentage of sperm with a curvilinear velocity > 10 μm/sec); (b) rapid sperm (RAP), as the percentage of sperm with a curvilinear velocity > 100 μm/sec); (c) curvilinear velocity (VCL, μm/sec); (d) amplitude of lateral head displacement (ALH, μm); and (e) beat-cross frequency (BCF, Hz). FNSW was used as a negative control, while scaled concentrations of cadmium were used for the positive control.

### Statistical analysis

All experiments were repeated three times in triplicate, and data were recorded as the mean with standard deviation (SD). For crustaceans, the 24, 48, 72 and 96 h median lethal concentration values (LC_50_) and related 95% confidence limits were calculated using the Litchfield–Wilcoxon method. For *P. lividus*, the median effective concentrations (EC_50_) were determined using the Trimmed Spearman-Karber statistical method. Prior to processing, all data were adjusted according to Abbott’s formula (ASTM 2004). The one-way analysis of variance (ANOVA) was used to detect statistical significance of the differences between the control and treated groups, and post hoc Tukey’s test was used to discriminate between results’ pair. When data failed to meet the assumption of normality and homoscedasticity, the non-parametric Kruskal–Wallis test was used to compare individual treatments. A two-way ANOVA with Tukey’s post hoc test was applied to detect significant effects of the parameter time (exposure period) and concentration (*p* < 0.05). All statistical analyses were conducted using Past3 Version 1.0 software.

## Results

### Toxicity to crustaceans

The negative controls showed mortality < 10% for both species. The 96h-LC_50_ values for ZnO NPs exposure were 0.37 and 0.30 mg/L for *C. truncata* and *G. aequicauda*, respectively*.* The exposure to ZnSO_4_ produced significantly higher LC_50_ values for *C. truncata* (0.63 ± 0.09 mg/L) than for *G. aequicauda* (0.28 ± 0.04 mg/L) (*p* < 0.05) (Table 2). Nevertheless, no significant differences between ZnO NPs and ZnSO_4_ toxicity were found between testing species at the considered exposure times.

**Table 2:** LC_50_ values (mg/L) for *C. truncata*, *G. aequicauda* and EC_50_ *P. lividus* embryos exposed to ZnO NPs and ZnSO_4_ (as mean values ± standard deviation; *n* = 3 per species per Zn form)

Endpoints	Time	Compounds	*C. truncata*	*G. aequicauda*	*P. lividus*
LC_50_	24 h	ZnO NPs	0.95 ± 0.11	n.a.	n.a.
		ZnSO_4_	1.37 ± 0.15	n.a.	n.a.
LC_50_	48 h	ZnO NPs	0.74 ± 0.16	0.76 ± 0.04	n.a. n.a.
		ZnSO_4_	1.07 ± 0.05	0.78 ± 0.14	
LC_50_/EC_50_	72 h	ZnO NPs	0.56 ± 0.22	0.40 ± 0.04	0.04 ± 0.008
		ZnSO_4_	0.80 ± 0.11	0.39 ± 0.06	0.06 ± 0.003
LC_50_	96 h	ZnO NPs	0.37 ± 0.07	0.30 ± 0.06	n.a. n.a.
		ZnSO_4_	0.63 ± 0.09	0.28 ± 0.04	

n.a., not available

**Fig. 1:**
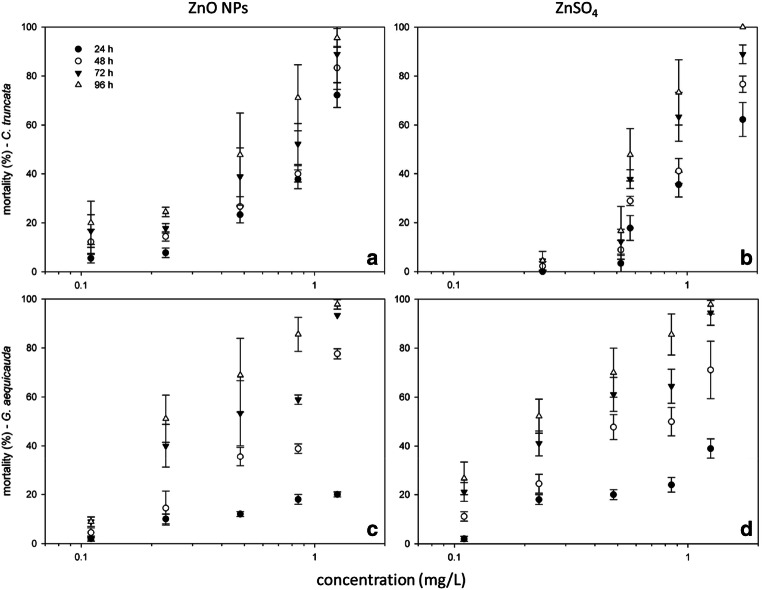
Relationship between concentration and mortality in *C. truncata* (**a** and **b**) and *G. aequicauda* (**c** and **d**) exposed to ZnO NPs or ZnSO_4_ for 24 to 96 h (as mean mortality values ± standard deviation; *n* = 9 per species and per Zn form; post hoc Tukey’s test results in Table S1, Supplemental Material); data were normalized to negative controls

The concentration–response curves at different exposure times (Fig. 1) for both crustaceans (see also Tables S1–S2, Supplementary Materials, summarizing the results of a two-way ANOVA on exposure time and concentration) evidenced that mortality increased with increasing exposure time for both ZnO NPs and ZnSO_4_. Both species showed similar mortality trends after exposure to both Zn forms, although ZnSO_4_ seemed a little more toxic than ZnO NPs. After 96-h exposure, *C. truncata* seemed less susceptible to ZnO NPs at the lowest concentrations tested, although *G. aequicauda* showed very low mortality at the shortest time exposure (24 h). In this species, the exposure time seemed more important than in *C. truncata*, with the mortality rates quite different at different times. Nevertheless, at the highest concentration after 96-h exposure, mortality was approximately 100% in all experimental batches.

### Toxicity to echinoderm

**Fig. 2:**
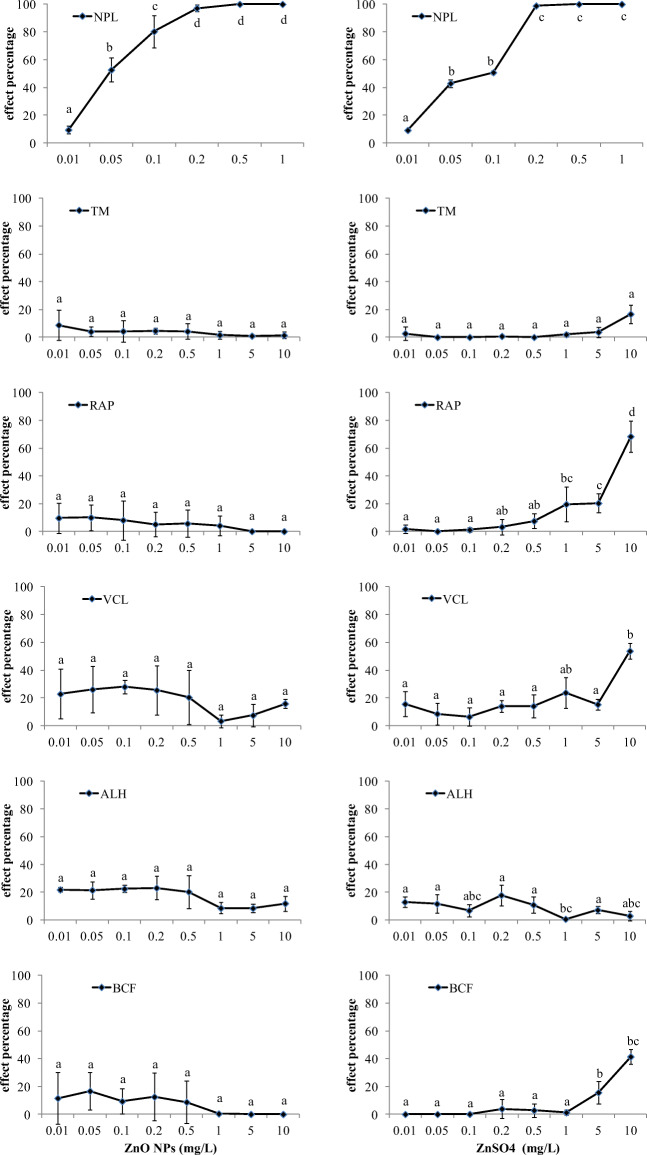
Percentage of effects (mean ± standard deviation; *n* = 3) on a multi-endpoint basis (normal developed plutei larvae (NPL), total motile sperm (TM), rapid sperm (RAP), curvilinear velocity (VCL), lateral head displacement (ALH) and beat-cross frequency (BCF)) in *P. lividus* sperm cells and embryos exposed to ZnO NPs and ZnSO_4_. Data with different letters (**a**–**d**) are significantly different (Tukey’s test, *p* < 0.05); data were normalized to negative controls

The effects of echinoderm sperm and embryo exposure to ZnO NPs and ZnSO_4_ on a multiple endpoints basis are shown in Fig. 2. Normal development of plutei larvae (NPL), total motility of sperms (TM), entity of rapid sperm population (RAP), curvilinear velocity (VCL), lateral head displacement (ALH) and beat-cross frequency of flagellum (BCF) are reported.

A clear dose–response was observed in the embryotoxicity test (NPL endpoint), where a 100% alteration was found at concentrations ≥ 0.2 mg/L for both ZnO NPs and ZnSO_4_, with calculated EC_50_ of 0.04 ± 0.008 mg/L and 0.06 ± 0.03 mg/L, respectively.

Conversely, none of the multi-endpoints of the motility parameters (MOT-tests) showed a clear concentration–effect response; hence, EC_50_ was not calculated. In detail, no adverse effects on the percentage of motile sperm (TM) or on the consistence of the sub-population of rapid sperm (RAP) were observed after 1 h of exposure to ZnO NP suspensions up to 10 mg/L; nevertheless, toxic effects were observed for the VCL endpoint (up to 40%) and for the ALH and BCF ones (up to 20%), although the Tukey’s post hoc tests did not highlight significant differences among concentrations. Similarly, the exposure to ZnSO_4_ up to 5 mg/L scarcely affected all the motility endpoints (always < 20%), although significant differences among ZnSO_4_ concentration were observed. On the contrary, the sperm cell suspensions exposed to 10 mg/L ZnSO_4_ highlighted a significant reduction of RAP, VCL and BCF endpoints (70, 55 and 45%, respectively) but no effect on TM and ALH ones.

## Discussion and conclusions

The bioassays with *C. truncata* and *G. aequicauda* assessed ZnO NP toxicity in comparison to ZnSO_4_. The bioassay with *C. truncata* is brand new and demonstrated its effectiveness to evaluate NPs toxicity. These results did not highlight significant differences between the two Zn forms but between the two species of crustaceans. The bioassay on *P. lividus* embryos did not show differences between the two Zn forms, both eliciting quite high toxicity. Hence, the order of decreasing toxicity for both ZnO NPs and ZnSO_4_ obtained from this study is *P. lividus* > *G. aequicauda* ≈ *C. truncata*. The comparable toxicity of ZnO NPs and its bulk form can be imputed to the high dissolution rate of ZnO NPs (46%; see Chemical analysis and NP characterization) as already demonstrated by Prato et al. (2020) with direct measure of the concentration of dissolved Zn ions in the experimental medium or in filtered ZnO NP suspensions and the findings of comparable toxicity of ZnO NPs and the relative bulk forms (ZnCl_2_ or ZnSO_4_). Prato et al. (2020) stated that the dissolution of ZnO NPs in FNSW depended on their concentrations. Our experimental design included a range of concentrations of ZnO NPs that can be deemed as rapidly and completely solubilized. Data are in agreement with those of Fairbairn et al. (2011), showing an almost complete dissolution within 10–20 h at 0.1 and 1 mg/L, although at higher ZnO NP concentration (10 mg/L), a slower dissolution rate was found (i.e. after the first 24 h) (Fairbairn et al. 2011). The contribution of dissolved Zn^2+^ ions to the ecotoxicity of ZnO NPs is considered pivotal by Aruoja et al. 2009; Blinova et al. 2010; Fairbairn et al. 2011; and Wong et al. 2010, ascribing the toxic effect of ZnO NPs mainly to the Zn^2+^ ion dissolution.

In the literature, we could not find references for ZnO NPs’ and ZnSO_4_ toxicity in the tested crustaceans; particularly, no data on *C. truncata* are available, and few studies utilized marine isopods as a model species in ecotoxicological study, although they are relevant models in soil ecotoxicology.

Data on ZnO NP toxicities are available for several other crustaceans: in the freshwater *D. magna* (Muna et al. 2019) (Heinlaan et al. 2008) calculated a 48 h LC_50_ of 3.2 and 2.6 mg/L, respectively; in the same studies, the LC_50_ for *T. platyurus* was calculated as 0.18 and 0.14 mg/L, respectively. Both studies demonstrated that Zn^2+^ ions were responsible of the ZnO NP toxicity, found in the test media at concentrations ranging from 40 μg/L to 58 mg/L. In the marine copepod *Tigriopus japonicus* and in the amphipod *Elasmopus rapax* nauplii, the ZnO NP ecotoxicity tests demonstrated that ZnO NPs (26.2 ± 5.1 nm, mean diameter) were more toxic to *T. japonicus* than to *E. rapax* (Wong et al. 2010). Furthermore, Fabrega et al. 2012) highlighted that the chronic exposure to ZnO NPs (35 ± 10 nm, mean diameter at 1 mg/L) can significantly affect the survival, growth and reproduction of *Corophium volutator*. It is worth to note that toxicity data of ZnO NPs and ZnSO_4_ are often based only on nominal concentrations (Vimercati et al. 2020), without taking into consideration the possible different effects of the high ionic strength of the saline medium on NPs (in the case of marine tests) as well as their sedimentation rate or shielding effect (i.e. reduction of the repulsive force among NPs due to their relative charge) (Rotini et al. 2017).

Due to the rapid and wide solubilization of ZnO NPs at the concentrations used in this study, the sea urchin sperms and embryos have been exposed to solubilized Zn^2+^ throughout the entire assay, and, consequently, the toxicity of ZnO NPs may be largely attributed to the dissolution of Zn ions into seawater. Motile sperm tests did not show a clear concentration–response when exposed to ZnO NP suspensions, only VCL (curvilinear velocity) being slightly affected by the exposure, while the other endpoints as lateral head displacement (ALH, depending on active sperm motility) and beat-cross frequency (BCF, depending on the flagellar beating) (Gallego et al. 2014; van der Horst et al. 2018) seemed not affected, indicating no clear alteration of both the structural integrity of the sperm cell and to the physiology of sperm movement.

The results obtained in this study are in agreement with those of Ozgur et al. (2018), who found that *C. carpio* spermatozoa exposed to ZnO NPs were slightly affected, with significant alteration only on VCL and BCF. The limited effect of ZnO NPs and ZnSO_4_ on *P. lividus* sperm motility is also consistent with the slight effect on fertilization found by Manzo et al. (2013) for the same species. The exposure of sea urchin embryos to ZnO NPs or ZnSO_4_ produced similar EC_50_ values *(p* > 0.05), further involving ionic zinc as responsible of the toxicity. The results on embryotoxicity agree with previous studies. Manzo et al. (2013) found 100% of *P. lividus* abnormal plutei larvae after exposure to 0.06-mg/L ZnO NPs, and Fairbairn et al. (2011) found 100% of abnormal plutei in *Lytechinus pictus* exposed to 0.15 mg/L ZnO NPs. Mos et al. (2017) and Smital and Kurelec (1998) observed only 10% of normal gastrulae in *Tripneustes gratilla* exposed to 0.1-mg/L ZnO NPs. Similarly, the EC_50_ values in *P. lividus* embryos exposed to ZnSO_4_ are comparable to those found for *L. pictus* (Fairbairn et al. 2011) and for *P. lividus* (Arizzi Novelli et al. 2003). Soluble Zn ions can cause abnormal morphology in embryos of the sea urchin *L. pictus* embryos after 96 h (Fairbairn et al. 2011), but they also hindered growth, delaying sexual maturation and affecting reproduction in the amphipod *C. volutator* at concentrations ranging between 0.2 and 1 mg/L under chronic exposure (100 days) (Fabrega et al. 2012). Conversely, Zhu et al. (2009) observed lower toxicity of Zn^2+^ than ZnO NPs on *Danio rerio* embryos and stated that ZnO NPs may produce more ROS than Zn^2+^ at least in freshwater impairing the antioxidant defence system. This hypothesis has been confirmed on different organisms, the marine algae *Thalassiosira pseudonana*, *Skeletonema marinoi*, *Dunaliella tertiolecta* and *Isochrysis galbana* by Miller et al. (2010) who imputed the toxic effect of ZnO NPs to the adsorption of particles onto the cell surface, damaged by both the NP production of ROS and the dissolved of Zn ions. Hence, the toxic effects of ZnO NPs cannot be only explained by the zinc ion action, but they must be linked to the physicochemical nature of the nanoparticles, such as their size. For example, at a concentration of 100 mg/L, ZnO NPs of 10–30 nm size are significantly more toxic than ZnO NPs of 200 nm to the brine shrimp *Artemia salina* (26% vs 18% mortality; after 96 h) (Ates et al. 2013; Libralato 2014). As a last point, the toxicity of ZnO NPs might be also caused by mechanical injury to the organisms, as happens in the marine copepod *T. japonicus* damaged by ZnO NP aggregates attached to the exoskeleton (Wong et al. 2010).

In conclusion, this study demonstrated the toxicity of ZnO NPs (according to concentration and exposure time) towards two marine crustaceans, *C. truncata* and *G. aequicauda*, and their feasibility as model species for NP studies. Toxicity has been found comparable to ZnSO_4_ one, suggesting a role of dissolved Zn ions in the toxicity of ZnO NP, although other possible mechanisms of toxicity for the NPs have to be considered. Furthermore, being isopods relevant model organisms in other environmental compartment, the newly tested species, *C. truncata*, seemed to present promising characteristics as a new potential easy-to-use testing species. Further focused studies should be carried out about. The study also highlighted the ecotoxicity of ZnO NPs to the sperm and developing embryos of the sea urchin *P. lividus*. Although a direct quantification of ZnO NPs in the marine environments is difficult to obtain, according to model-based estimates, it should range from hundreds ng/L to about 900 μg/L (Yung et al. 2015); therefore, the effects on different developmental endpoints even at low concentrations highlight the toxicity of these contaminants to sea urchin embryos, probably elicited also in the field.

This study prompts the urgency of monitoring ZnO NPs in the marine environment because of their potential effects on sensitive species providing a first set of data about concentration– and time–response relationships. Special attention should be devoted to the molecular mechanisms of the considered biological models to better investigate the action of both ZnO NPs and their dissolved forms, as already done for CuO NPs (Gallo et al. 2018). This may help the understanding of ZnO NPs’ environmental implications for the saltwater compartment supporting the process for the identification of environmental quality benchmarks.

## Supplementary Information

ESM 1 (DOCX 18 kb).

## Data Availability

All data generated or analysed during this study are included in this published article and its supplementary information files.
