# Supplementary material for: Comparative toxicity of ionic and nanoparticulate zinc in the species Cymodoce truncata, Gammarus aequicauda and Paracentrotus lividus
Source: Environ Sci Pollut Res Int. 2021 Apr 7;28(31):42891–900. doi: 10.1007/s11356-021-13712-0 (PMC8354894; doi:10.1007/s11356-021-13712-0)
Supplement: Supplementary file 1 — (DOCX 18 kb). [file 11356_2021_13712_MOESM1_ESM.docx]

Supplementary Materials

Table S1. Post-hoc Tukey’s test results. Data with different letters (a–d) are significantly different (Tukey’s test, p < 0.05)

| *C. truncata* |  | Time (h) | CTR | 0,1 | 0,25 | 0,5 | 1 | 2 |
| --- | --- | --- | --- | --- | --- | --- | --- | --- |
|  | *ZnO NPs* | 24 | a | ab | b | c | d | e |
|  |  | 48 | a | b | b | c | d | e |
|  |  | 72 | a | b | b | c | c | d |
|  |  | 96 | a | b | b | c | d | e |
|  |  |  |  |  |  |  |  |  |
|  | *ZnSO_4_* | 24 | a | a | a | b | c | d |
|  |  | 48 | a | a | b | c | d | e |
|  |  | 72 | a | ab | b | c | d | e |
|  |  | 96 | a | a | a | b | c | d |
| *G. aequicauda* |  |  |  |  |  |  |  |  |
|  | *ZnO NPs* | 24 | a | a | ab | bc | cd | d |
|  |  | 48 | a | a | b | c | c | d |
|  |  | 72 | a | a | b | c | c | d |
|  |  | 96 | a | a | b | c | cd | d |
|  |  |  |  |  |  |  |  |  |
|  | *ZnSO_4_* | 24 | a | a | ab | b | b | c |
|  |  | 48 | a | a | b | c | c | d |
|  |  | 72 | a | b | c | d | d | e |
|  |  | 96 | a | b | c | d | e | e |

Table S2. Results of two-way ANOVA performed on mortality data (%) of each test species at each concentration and all exposure time. Bold value indicate significance at p<0.05.

|  | ZnO NPs | | | | ZnSO_4_ | | | |
| --- | --- | --- | --- | --- | --- | --- | --- | --- |
| Source | df | Mean square | *F-ratio* | *p* | df | Mean square | *F-ratio* | *p* |
| *C. truncata* |  |  |  |  |  |  |  |  |
| Concentration | 4 | 10304.4 | 180.1 | 0.000 | 4 | 12554.9 | 335.6 | 0.000 |
| Time | 3 | 1422.72 | 24.86 | 0.000 | 3 | 1760.68 | 47.07 | 0.000 |
| Conc x Time | 12 | 57.4383 | 100 | 0.4663 | 12 | 148.488 | 3.969 | 0.000 |
| Within | 40 | 57.2222 |  |  | 40 | 374.074 |  |  |
|  |  |  |  |  |  |  |  |  |
| *G. aequicauda* |  |  |  |  |  |  |  |  |
| Concentration | 4 | 7241.07 | 160.9 | 0.000 | 4 | 6342 | 132.7 | 0.000 |
| Time | 3 | 7236.11 | 160.8 | 0.000 | 3 | 6145.92 | 128.6 | 0.000 |
| Conc x Time | 12 | 542.98 | 12.07 | 0.000 | 12 | 162.678 | 3.405 | 0.002 |
| Within | 40 | 44.9998 |  |  | 40 | 47.7776 |  |  |
